# The impact of public health shocks on fertility intentions: evidence from the COVID-19 pandemic in China

**DOI:** 10.3389/fpubh.2025.1631821

**Published:** 2025-08-18

**Authors:** Yiting Guo, Yuqi Mou, Yan Peng, Chen Zhang

**Affiliations:** ^1^Economics and Management School, Wuhan University, Wuhan, China; ^2^The Wang Yanan Institute for Studies in Economics, Xiamen University, Xiamen, China; ^3^School of Economics and Management, Huangshan University, Huangshan, China

**Keywords:** fertility intentions, COVID-19, public health shocks, difference-in-differences, internet search index data

## Abstract

**Background:**

China's persistent fertility decline poses serious long-term demographic and socioeconomic challenges. The COVID-19 pandemic has introduced additional uncertainty, raising questions about how external shocks affect fertility intentions in real time.

**Objective:**

This study examines the causal impact of localized COVID-19 shocks on fertility intentions in China, as measured by high-frequency digital search data that capture real-time behavioral shifts.

**Methods:**

We construct a monthly city-level fertility index based on Baidu search volumes for pregnancy-related keywords across 222 cities (2019–2022). COVID-19 exposure is measured using sustained “high-risk” status over 14 consecutive days. A staggered difference-in-differences design is employed, with robustness checks including imputation-based estimators, event-study analysis, and heterogeneity analysis by city characteristics.

**Results:**

COVID-19 shocks led to a significant 5.4% decline (*p* < 0.01) in fertility-related search activity across Chinese prefecture-level cities. Event study confirmed persistent post-shock suppression, while placebo simulations confirmed the robustness of the identification strategy. Heterogeneity analysis revealed stronger declines in cities with higher GDP per capita (*p* < 0.01), greater urbanization (*p* < 0.01), and larger female population shares (*p* < 0.01), highlighting the amplifying role of local socioeconomic conditions.

**Conclusion:**

Fertility intentions respond sharply to pandemic-related uncertainty, especially under pressure from economic and institutional constraints. The findings underscore the fragility of reproductive intentions under uncertainty and highlight the importance of tailoring fertility policy to local socioeconomic environments.

## 1 Introduction

Fertility rates are not merely demographic outcomes but integral components of long-term economic performance, innovation, and public welfare sustainability (1–4). Especially for a populous country like China, fertility plays an important role in the macroeconomic system—it is shaped by economic factors such as income uncertainty, housing costs, and social mobility, while also exerting feedback effects on growth, labor markets, and welfare systems (5–7). Between 2000 and 2015, China's total fertility rate (TFR) declined from 1.42 to 1.05. Although a nationwide two-child policy introduced in 2016 temporarily raised the TFR to 1.59 by 2017, the rebound was short-lived, and the 2021 three-child policy similarly failed to reverse the downward trend. By 2022, China's TFR had returned to 1.05, ranking fifth lowest globally, below both the United States (1.62) and Japan (1.20)^1^. These patterns highlight the fragility of fertility intentions in China. Beyond social structural and policy determinants, episodic disruptions–especially those related to public health—may further constrain reproductive behavior. This raises urgent questions about how external shocks—most notably the COVID-19 pandemic—have influenced fertility behavior in real time.

Unlike macroeconomic downturns, public health shocks influence fertility through a more diverse set of channels, combining behavioral responses to perceived health risks with disruptions in public services and social infrastructure. These include heightened uncertainty, increased caregiving burdens, and reduced access to reproductive services—all of which may lead households to delay or forgo childbearing. Although many of these mechanisms are difficult to observe directly, real-time shifts in fertility-related behavior may offer early signals of changing reproductive intentions, particularly in the absence of timely demographic data.

Among external shocks to demographic behavior and household decision-making, the COVID-19 pandemic stands out as an unprecedented global crisis. It not only disrupted economic systems, healthcare infrastructure, and social life, but also had profound effects on fertility behavior—including changes in reproductive intentions, timing of childbearing, and family size preferences–alongside broader population dynamics^2^. Globally, the demographic consequences of COVID-19 have become a central focus of social science research (10, 11). In China, where fertility rates were already under pressure from economic and institutional challenges, the pandemic further exacerbated these pressures: job instability, high childcare costs, and rising uncertainty have led many families to postpone or abandon childbearing plans. Although some anticipated a “lockdown baby boom” due to increased time at home and reduced leisure alternatives, empirical evidence suggests the opposite (12). Understanding the behavioral consequences of COVID-19 for fertility in China is therefore essential—not only for academic insight, but also for informing long-term, context-sensitive fertility-support policies. While prior studies have examined the economic and psychological impacts of COVID-19, limited evidence exists on how such shocks affect fertility intentions in near real time–particularly in the context of developing countries like China, where economic and institutional constraints on childbearing are already severe.

Prior research on fertility behavior has relied on diverse data sources–including census records, household surveys, and administrative birth statistics–to examine how reproductive decisions are shaped by economic conditions, gender norms, and institutional settings (13–15). While these studies have yielded valuable insights into the determinants of fertility, their dependence on traditional data sources often limits both temporal resolution and responsiveness to rapidly unfolding events. In particular, the demographic effects of fast-moving crises such as pandemics are difficult to detect in real time using conventional datasets. Recent work has addressed this limitation by using monthly birth statistics from high-income countries to evaluate the short-term fertility impacts of COVID-19 (16–18). For instance, (16) analyzes data from 22 countries and documents significant drops in crude birth rates in parts of Southern Europe, underscoring the value of high-frequency data in capturing behavioral responses to public health shocks. However, such timely official data are not available in many developing countries, including China, where birth statistics are typically published only at annual frequencies, often with considerable delays–and in many cases, with incomplete coverage at the prefecture or city level. This study helps fill that gap by leveraging real-time internet search data and exploiting cross-city variation in pandemic exposure to estimate the causal effects of COVID-19 shocks on fertility intentions.

Against this backdrop, we examine whether COVID-19 shocks causally affected fertility intentions in China. To overcome the limitations of delayed and coarse-grained birth statistics, we construct a monthly city-level fertility search index using Baidu Internet search data based on pregnancy-related keywords. Specifically, we track search volumes for four biomedical terms commonly used by individuals who are planning for or monitoring early-stage pregnancy: Human Chorionic Gonadotropin (HCG), Expected Date of Delivery (EDD), Progesterone, and Pregnenolone^3^. This behavioral proxy offers high-frequency insight into fertility intentions. These terms are commonly searched by individuals who suspect they might be pregnant, are actively preparing for conception, or are monitoring early pregnancy progress–thus capturing the anticipatory stage of fertility-related behavior. Unlike actual birth data, these search terms reflect early-stage reproductive behavior that is likely to translate into future fertility outcomes. We selected these terms for their medical specificity, semantic clarity, and consistent usage across regions. As standardized indicators in clinical practice throughout China, they reduce potential variation stemming from local medical protocols or sociocultural differences. Their appearance in search data typically signals concrete reproductive actions—such as undergoing hormone testing, seeking treatment for pregnancy maintenance, or confirming a due date—rather than general curiosity or engagement with broader discourse^4^. The analysis focuses on 222 prefecture-level cities with reliable internet penetration and consistent search activity. Leveraging the variation in the timing of cities entering “high-risk” COVID-19 status, we implement a staggered difference-in-differences design that accommodates non-synchronous treatment exposure across cities, using both two-way fixed effects with Goodman-Bacon decomposition (19) and the imputation-based estimator proposed Borusyak et al. (20).

While we do not directly observe realized fertility outcomes, our behavioral proxy reveals a substantial and persistent decline in fertility-related search activity following city-level COVID-19 shocks. These declines were not uniform: they were more pronounced in economically developed, highly urbanized areas, and in cities with a larger share of women in the population. This heterogeneity indicates that the behavioral responses to public health shocks vary systematically with local socioeconomic conditions, such as Economic development, urbanization, and gender composition. Moreover, the temporal pattern of decline–confirmed by event-study analysis–indicates that behavioral suppression persisted beyond the immediate period of shock exposure, rather than rebounding quickly. The results underscore the potential for sustained behavioral changes in fertility planning in the aftermath of large-scale public health shocks. By capturing these dynamics through high-frequency digital behavior, our study uncovers early signals of demographic stress that are not yet visible in conventional fertility statistics.

This study makes three core contributions to the literature on fertility behavior and public health shocks. First, we leverage high-frequency behavioral data from the Baidu Search Index as a novel proxy for fertility intentions of prefecture-level cities in China. Unlike traditional demographic statistics, which are delayed and aggregated, our approach captures near real-time fluctuations in reproductive attention, offering new insights into how family planning decisions dynamically respond to public health shocks. By shifting the analytical lens from realized births to behavioral signals, we uncover anticipatory responses that precede observable demographic outcomes–an aspect often missed in conventional fertility research. Second, we develop a granular and behaviorally salient measure of pandemic exposure, based on whether a city remained in “high-risk” status for 14 consecutive days. This indicator aligns with China's official risk classification system and captures localized disruptions–such as lockdowns, service suspensions, and healthcare strain–that plausibly influence household fertility decisions. Third, we address key identification challenges in evaluating asynchronous, city-level public health shocks by combining high-frequency behavioral data with a staggered difference-in-differences framework. This strategy accommodates variation in treatment timing and regional heterogeneity, and uncovers meaningful socioeconomic disparities in fertility responses. Specifically, we show that fertility suppression is significantly stronger in cities with higher income, greater urbanization, and a larger female population share. These findings underscore how socioeconomic context shapes the demographic consequences of public health crises, and highlight the importance of targeted, context-sensitive policy design.

Beyond empirical contributions, this study offers timely insights for policy. Facing persistent demographic headwinds, Chinese authorities and other low-fertility societies have increasingly shifted from short-term birth incentives toward structural reforms—such as subsidized childcare, gender-equal parental leave, and affordable education (21, 22). These changes reflect a broader recognition that fertility behavior is not solely a private matter, but is shaped by systemic factors–economic insecurity, caregiving burdens, and institutional capacity. By providing city-level evidence on how public health crises interact with these structural constraints, our findings inform the design of forward-looking demographic policy frameworks in aging and low-fertility societies.

Understanding how external shocks interact with city-level socioeconomic constraints is critical for designing resilient and forward-looking demographic policies. By providing city-level behavioral evidence on fertility responses to COVID-19, this study sheds light on how public health disruptions shape reproductive intentions in real time–well before such shifts materialize in official statistics. Moreover, our findings highlight the value of search-based behavioral data as a scalable and generalizable tool for population monitoring, particularly in low- and middle-income countries where timely demographic reporting is limited. These insights support the development of evidence-based fertility policy and contribute to global goals such as SDG 3 (Good Health and Well-being) and SDG 10 (Reduced Inequalities).

The remainder of the paper is organized as follows. Section 2 outlines the theoretical hypotheses. Section 3 describes the data sources, key variables, sample construction, and empirical strategy. Section 4 presents the main empirical results, including baseline estimates, imputation-based estimation, dynamic effects, and robustness analyses. Section 5 discusses key findings, theoretical implications, and policy relevance. Section 6 concludes.

## 2 Theoretical hypotheses

A substantial body of literature shows that fertility is shaped by a complex interplay of economic conditions, institutional support, social norms, psychological preferences, and family dynamics (23). Key determinants such as labor market conditions, housing affordability, access to childcare, and expectations about the future jointly influence when and whether individuals choose to have children, as well as their desired family size (24–28).

Historically, fertility tends to decline following major disruptive events such as wars, financial crises, and public health emergencies (29–31). These “negative shocks” affect fertility decisions through three primary channels: (1) heightened economic and employment uncertainty; (2) disrupted access to reproductive and health services; and (3) changes in social interaction, family roles, and psychological wellbeing (23, 32).

The COVID-19 pandemic is distinctive among such shocks in three important respects. First, its global simultaneity disrupted economic and health systems across nearly all regions. Second, its prolonged and uncertain trajectory—marked by repeated waves, lockdowns, and evolving policy responses—sustained a climate of insecurity. Third, it transformed everyday life through remote work, school closures, and service disruptions, which disproportionately increased the caregiving burden on families, particularly on women (10, 33). In addition, concerns over the possible impact of COVID-19 infection on fetal health may have contributed to delays in childbearing (31).

Empirical evidence on the pandemic's fertility impact remains mixed. Cross-national studies report short-term declines in birth rates in countries such as the United States, Italy, Spain and Australia (16, 34, 35), whereas others observe minimal changes or even slight rebounds-particularly in countries with generous welfare systems and effective policies in support of families and employment, such as Nordic countries like Norway (16, 36). At the micro level, survey-based studies find widespread postponement or cancellation of pregnancy plans, though some groups reported an increased desire for children–often associated with altered life priorities or increased couple time during lockdown (37, 38).

These divergent findings are likely driven by differences in outcome measures (e.g., realized births vs. stated intentions), timing (early vs. later stages of the pandemic), and contextual factors such as economic resilience and welfare regimes. A key limitation in the existing literature is the lack of high-frequency, localized indicators that can capture short-term fertility dynamics during public health crises–particularly in developing countries. Our study addresses this gap by leveraging city-level online search behavior in China, offering a timely and granular lens into how the pandemic influenced reproductive decision-making.

Drawing on the above literature and contextualizing within China's socioeconomic and policy environment, we hypothesize that exposure to COVID-19-related uncertainty suppresses short-term fertility planning. This behavioral adjustment is expected to manifest as a decline in online searches for pregnancy-related information, which serve as a timely proxy for fertility intentions. Accordingly, we propose Hypothesis 1.

Hypothesis 1. **Main Effect:** Exposure to COVID-19 shocks is negatively associated with fertility-related search behaviors.

We further expect that this relationship varies systematically across cities, reflecting underlying city-level heterogeneity. Drawing on prior work, we focus on three key city-level characteristics: economic development, urbanization, and gender composition. Accordingly, we propose Hypothesis 2.

Hypothesis 2. **Heterogeneity:** The impact of COVID-19 shocks on fertility-related behavior varies by city-level socioeconomic characteristics.

We now detail each in turn. First, cities with higher levels of economic development may experience stronger negative fertility responses. These cities typically have lower baseline fertility and higher opportunity costs of childbearing. Residents are more sensitive to labor market shocks and more reliant on formal childcare infrastructure–both of which were severely disrupted by the pandemic (10, 23). Accordingly, we propose Hypothsis 2.1.

Hypothesis 2.1. **Economic development:** The negative impact of COVID-19 shocks on fertility-related behavior is stronger in economically developed cities.

Second, urbanization likely amplifies fertility suppression. Urban areas tend to have delayed family formation, higher living costs, and more competitive labor markets. During the pandemic, they also experienced stricter lockdowns, longer school closures, and more acute service interruptions—factors that jointly constrained reproductive behavior (17, 26).

Moreover, urban and rural areas experienced the pandemic in structurally different ways. Urban residents faced greater mobility restrictions and emotional strain, whereas rural areas–initially less affected—faced surges later, often with weaker institutional responses (39, 40). On balance, however, urban areas bore the brunt of prolonged disruption, with stronger potential impacts on fertility intentions. Accordingly, we propose Hypothsis 2.2.

Hypothesis 2.2. **Urbanization:** The negative impact of COVID-19 shocks on fertility-related behavior is more pronounced in highly urbanized areas.

Third, gender composition may moderate fertility responses. Cities with a higher female population share may experience larger declines in fertility-related behavior, as women disproportionately shoulder caregiving burdens—including childcare, education, and older adult care—particularly during periods of crisis. Pandemic-related disruptions to female employment and heightened household stress are also likely to contribute to postponed or foregone childbearing plans (33, 41).

Importantly, a city with a higher proportion of women often corresponds to greater female social and economic empowerment. In the Chinese context, such regions tend to exhibit stronger female labor market participation and more equitable household decision-making structures. These characteristics amplify the behavioral impact of caregiving burdens, as women not only absorb a larger share of crisis-induced responsibilities, but also play a decisive role in fertility planning. Supporting this, recent micro-level evidence shows that Chinese women hold substantial influence over decisions about whether and when to have (additional) children, especially under conditions of uncertainty or constraint (42). Together, these dynamics suggest that pandemic-related disruptions may have translated more directly into suppressed fertility intentions in cities with a higher female population share. We therefore propose Hypothesis 2.3.

Hypothesis 2.3. **Gender Composition:** Cities with a higher female population share experience larger declines in fertility-related behavior.

## 3 Methods

### 3.1 Data and variables

#### 3.1.1 Dependent variable

Official birth data in China are published annually and often lack granularity at the city level, making it difficult to analyze short-term fertility dynamics, especially during rapidly evolving events such as the COVID-19 pandemic. To address this limitation, we construct a high-frequency proxy for fertility-related behavior using Baidu Search Index data^5^, which tracks keyword search volumes across both mobile and PC platforms.

Baidu is the dominant search engine in China, with a market share exceeding 90% (43). Functionally analogous to Google Trends used in other countries, Baidu Index offers a widely accepted proxy for gauging population-level interest and behavioral patterns. Its broad user base and responsiveness to real-world events make it a reliable indicator for real-time analysis (44).

We collect daily Baidu Search Index data for four fertility-related keywords–*Human Chorionic Gonadotropin (HCG), Expected Date of Delivery (EDD), Progesterone*, and *Pregnenolone*—from both mobile and PC platforms. These terms are tightly associated with different stages of early pregnancy:

- *HCG* is a critical biomarker for early pregnancy confirmation (45).
- *EDD* searches reflect pregnancy planning following confirmation.
- *Progesterone* and *Pregnenolone* are essential hormones for maintaining early-stage pregnancy (46, 47).

We construct a composite fertility search index for each city-day observation by taking the simple average of the search volumes of four pregnancy-related terms. This index (*Index*) provides a real-time, high-frequency measure of fertility-related behavior. Given the need to smooth daily volatility and mitigate short-term noise, our main empirical analysis relies on monthly aggregates, calculated by averaging the daily index across all available days within each city-month. For robustness, we also examine alternative temporal resolutions, including quarterly, weekly, and daily frequencies. To enhance transparency and address concerns about data quality, we report in Supplementary Table S2 the proportion of zero values for each keyword and for the composite search index across multiple temporal resolutions (daily, weekly, monthly, and quarterly). We emphasize that no imputation, interpolation, or exclusion of zero values was performed; all raw data were retained to preserve the integrity of the original Baidu search signals. Although zero values are relatively common in the raw daily data–especially for individual keywords–their prevalence declines substantially with temporal aggregation. At the monthly level, which is used in our main analysis, the proportion of zeros falls below 5% for all variables, and drops to just 1.2% for the composite index. These results support the robustness and reliability of our search-based proxy. The index reflects behavioral intentions and information-seeking related to pregnancy, and is widely regarded as a leading indicator of fertility-related decision-making (48).

The fertility search index dataset covers 333 prefecture-level cities from January 1, 2019, to December 31, 2022, providing broad geographic coverage and allowing for the analysis of fertility-related patterns before, during, and after the COVID-19 pandemic.

While online search activity reflects information-seeking behavior rather than finalized decisions, a growing body of demographic research supports its use as a timely proxy for fertility intentions. For instance, (49) demonstrate that Google search volumes for pregnancy-related terms closely predicted the subsequent decline and recovery of U.S. births during the COVID-19 pandemic. Similarly, (50) and (51) show that fertility-related searches correlate strongly with regional and socioeconomic variations in birth patterns across the U.S. and Europe. These studies suggest that aggregated search behavior offers meaningful signals of fertility planning, especially under conditions of uncertainty when conventional data are unavailable or delayed.

We further assess the validity of this search-based proxy in Section 1 of the Supplementary material, where we show that the fertility search index is positively and significantly associated with official city-level birth rates in 2019. The correlation remains robust after controlling for key socioeconomic factors, lending empirical support to the index's representativeness as a proxy for fertility-related behavior.

To further clarify the behavioral scope of our proxy, we note that the selected keywords—“HCG”, “EDD”, “Progesterone” and “Pregnenolone”—are tightly linked to early pregnancy monitoring and medical decision-making. These terms are unlikely to be frequently searched by the general population and are more relevant to individuals who are already pregnant or actively preparing for pregnancy. While search activity captures information-seeking rather than finalized decisions, we assume that incidental or curiosity-driven searches remain relatively stable over time. Consequently, observed shifts in aggregate search intensity are more likely to reflect behavioral adjustments among those with actual or imminent fertility considerations. In this sense, the search index provides a high-frequency, spatially granular signal of fertility-related intentions under uncertainty, which aligns with the central aim of our study.

#### 3.1.2 Key independent variable

The key independent variable is a city-level indicator capturing exposure to COVID-19 shocks. In accordance with national public health guidelines^6^. A city is defined as “treated” if it was either designated as high-risk for at least 14 consecutive days or placed under a government-mandated citywide lockdown.

Since official daily high-risk designations were not available before December 1, 2020, we apply a proxy definition based on the national guidelines issued in February 2020—classifying a city as high-risk if it reported more than 50 new cases over 14 consecutive days, using publicly available case counts from the National Government Service Platform^7^. From December 1, 2020, to December 22, 2022, we manually compiled daily high-risk area data based on official announcements from the National Government Service Platform and the health commissions of provincial and municipal governments. After December 22, 2022, China officially downgraded COVID-19 management from “Class B infectious disease managed as Class A” to standard “Class B management,” and the classification of high-risk and low-risk areas was discontinued; accordingly, our observation period ends on this date.

In China's institutional context, being designated as a high-risk area entailed immediate and strict public health interventions, such as localized lockdowns, travel restrictions, business closures, mass testing, and centralized quarantine. Therefore, high-risk classification serves as a credible proxy for the localized severity of the pandemic and the associated disruptions to daily life.

In line with this definition, cities that were ever designated as high-risk for 14 consecutive days or that implemented city-wide lockdowns are classified as the treatment group. Cities that neither reached high-risk status for that duration nor imposed lockdowns throughout the study period are assigned to the control group. It is worth noting that although a nationwide home quarantine policy was in place during the 2020 Spring Festival, many cities did not experience local outbreaks or implement additional epidemic control measures beyond general stay-at-home recommendations. These cities are considered unaffected by direct pandemic shocks and are thus included in the control group.

For cities in the treatment group, the timing of exposure (i.e., the “treatment point”) is determined based on when they met the criteria for high-risk classification or imposed strict lockdowns. Due to incomplete data coverage in the early stages of the pandemic, we treat January 23, 2020–the date of Wuhan's city-wide lockdown–as the treatment onset for all cities in Hubei Province, reflecting the widespread impact of the outbreak in the region.

Specifically, for each treated city, we identify the first calendar month in which the 14-day high-risk threshold was met or a citywide lockdown was imposed, and assign *Shock*_*ct*_ = 1 for that month and all subsequent months in our baseline analysis. For control cities, *Shock*_*ct*_ remains 0 throughout the entire study period, where *c* indexes cities and *t* denotes months. The definition of this key explanatory variable is summarized in Panel B of Table 1.

**Table 1:** Variable definitions.

**Variable name**	**Variable description**	**Unit**
**Panel A: dependent variables**		
*HCG*	Baidu Search Index for HCG	-
*EDD*	Baidu Search Index for expected date of delivery	-
*Progesterone*	Baidu Search Index for progesterone	-
*Pregnenolone*	Baidu Search Index for pregnenolone	-
*Index*	Average of the four fertility search indices	-
**Panel B: key independent variable**		
*Shock*_*ct*_	COVID-19 shock city: 1 if city *c* at time *t* is exposed to pandemic shock (was designated as a high-risk area), 0 otherwise	-
**Panel C: control variables**		
*perGDP*	GDP per capita	Yuan
*UrbanRate*	Urban population / total population × 100	%
*UnempRate*	Registered unemployment rate × 100	%
*MFgender*_*ratio*	Male population / female population × 100	%
*Edu*	Average years of education	Years

#### 3.1.3 Control variables

To adjust for baseline differences across cities, we include a set of annual control variables that are plausibly associated with fertility behavior. Specifically, we control for GDP per capita (*perGDP*), urbanization rate (*UrbanRate*), unemployment rate (*UnemploymentRate*), and male-to-female ratio (*MFgender*_*ratio*), all drawn from the China City Statistical Yearbook. These variables capture key aspects of economic development, demographic structure, and labor market conditions, helping to ensure that unobserved heterogeneity does not confound the estimated effects of COVID-19 shocks. To reduce skewness and improve comparability across cities, we apply a logarithmic transformation to GDP per capita. Definitions of all variables are provided in Panel C of Table 1.

#### 3.1.4 Sample selection

To ensure data quality and regional comparability, we restrict the sample to cities located east of the Hu Huanyong Line—a widely recognized demographic and economic divide in China—resulting in 253 out of 333 prefecture-level cities (52). This restriction, which excludes six western provinces (Xinjiang, Tibet, Gansu, Qinghai, Ningxia, and Inner Mongolia), follows established practice in empirical research (53–55), given the substantially lower population density and internet penetration rates in these regions.

After excluding cities with missing values in control variables, the final analytical sample consists of 222 prefecture-level cities. Among them, 156 cities are classified as the treatment group (exposed to COVID-19 shocks), while the remaining 66 cities form the control group (unexposed throughout the study period).

Table 2 presents descriptive statistics for the main variables, separately for treated and untreated cities. Cities in the treatment group exhibit higher search volumes for fertility-related keywords, along with higher levels of economic development and urbanization. These differences reflect the fact that COVID-19 outbreaks were more likely to occur in economically active and densely populated urban centers. In our empirical analysis, we control for these baseline characteristics to mitigate potential confounding and enhance the comparability between the two groups.

**Table 2:** Descriptive statistics of variables.

**Variable (mean)**	**Not shocked cities (*N* = 66)**	**Shocked cities (*N* = 156)**	**Overall (*N* = 222)**
**Panel A: dependent variables**			
*HCG*	35.42	68.40	58.60
*EDD*	30.37	56.96	49.05
*Progesterone*	42.50	75.44	65.64
*Pregnenolone*	33.63	60.62	52.59
*Index*	35.48	65.40	56.50
**Panel B: control variables**			
ln(perGDP)	10.81	10.94	10.90
*UrbanRate*	53.08	57.40	56.11
*UnempRate*	2.66	2.76	2.73
*MFgender*_*ratio*	105.98	104.37	104.85

### 3.2 Empirical strategy

We adopt a Difference-in-Differences (DID) framework with two-way fixed effects to estimate the causal effect of COVID-19 shocks on fertility-related outcomes. The empirical model is formally specified in Equation 1.


(1) $$\begin{array}{c} Y_{ct}=\alpha +\beta Shock_{ct}+X_{c}\mu_{t}+\lambda_{c}+\mu_{t}+\epsilon_{ct} \end{array}$$

where *Y*_*ct*_ represents the fertility-related outcome for city *c* at time *t*, including the five fertility proxies listed in Panel A of Table 1. The key independent variable *Shock*_*ct*_ is a binary indicator equal to 1 if city *c* has been exposed to a COVID-19 shock at time *t*, and 0 otherwise. The term *X*_*c*_μ_*t*_ represents the interaction between city-level control variables *X*_*c*_ from 2019 (prior to the pandemic) and time fixed effects μ_*t*_, allowing the effect of baseline characteristics to vary across time. City fixed effects (λ_*c*_) absorb time-invariant unobserved heterogeneity such as geographic or cultural factors, while time fixed effects (μ_*t*_) capture nationwide shocks affecting all cities, such as changes in national policies. The error term ϵ_*ct*_ captures idiosyncratic variation. The coefficient of interest, β, captures the average treatment effect of COVID-19 shocks on fertility outcomes. To address potential serial correlation in the error terms, we cluster standard errors at the city level throughout the analysis.

## 4 Results

We begin by presenting baseline estimates from a two-way fixed effects model. To assess the credibility of the identification strategy, we next conduct a Goodman-Bacon decomposition and implement an imputation-based estimator designed for settings with staggered treatment timing. We then examine the dynamic effects of COVID-19 shocks using an event study framework and perform placebo tests to address concerns about spurious associations. To further verify robustness, we replicate the analysis using alternative temporal frequencies–quarterly, weekly, and daily. Finally, we explore heterogeneity in treatment effects across cities with varying levels of economic development, urbanization, and demographic structure.

### 4.1 Baseline estimates using two-way fixed effects

Given the extended duration of the study and the high frequency of the underlying data, we aggregate daily fertility-related search indices into monthly averages from January 2019 to December 2022, resulting in a balanced panel with 48 monthly observations per city. Our baseline estimation adopts a standard two-way fixed effects (TWFE) specification with city and month fixed effects, estimated using ordinary least squares (OLS). To account for pre-existing cross-sectional differences, we include interaction terms between 2019 city-level characteristics and month fixed effects. The control variables consist of the logarithm of GDP per capita, the male-to-female ratio, the urbanization rate, and the registered unemployment rate.

Table 3 reports the estimated effects of COVID-19 shocks on each of the five fertility-related search indices. Columns (1) through (4) present results using individual keywords–HCG, EDD, Progesterone, and Pregnenolone–as dependent variables. Column (5) uses the composite index as the dependent variable. The coefficient on *Shock* in Column (5) is estimated at −3.546 and is statistically significant at the 1% level, indicating that exposure to a COVID-19 shock reduces the daily fertility search index by approximately 3.5 points. Given that the pre-treatment mean index value for treated cities is approximately 65.4 (see Table 2), this corresponds to a decline of about 5.4%. Similar negative and significant effects are observed across all individual indices. These estimates capture the average short-term response across cities with varying timing and intensity of exposure, net of fixed effects and baseline heterogeneity. The consistency of results across all four individual keywords and the composite index reinforces the robustness of our findings and suggests that the observed decline is not driven by any single term.

**Table 3:** The impact of COVID-19 shocks on fertility-related search index: two-way fixed effects analysis.

**Dependent variable**	**HCG**	**EDD**	**Progesterone**	**Pregnenolone**	**Index**
	**(1)**	**(2)**	**(3)**	**(4)**	**(5)**
Shock	-3.351***	-5.680***	-2.369*	-2.385**	-3.546***
	(1.115)	(1.538)	(0.918)	(1.072)	(1.074)
Constant	59.155***	50.054***	66.048***	53.006***	57.114***
	(0.189)	(0.261)	(0.156)	(0.182)	(0.182)
2019 city controls × Month FE	Yes	Yes	Yes	Yes	Yes
City FE	Yes	Yes	Yes	Yes	Yes
Month FE	Yes	Yes	Yes	Yes	Yes
Observations	10,656	10,656	10,656	10,656	10,656
Adjusted *R*^2^	0.972	0.945	0.971	0.959	0.976

Standard errors clustered at the city level are reported in parentheses. *p < 0.1, **p < 0.05, ***p < 0.01.

It is worth noting that the negative effects we identify likely represent a lower-bound estimate of the true behavioral suppression induced by COVID-19 shocks. Several factors contribute to this conservative estimate. First, in cities experiencing active outbreaks and classified as high-risk during the pandemic, strict containment measures may have severely limited access to in-person reproductive healthcare services, such as consultations with obstetricians or prenatal care providers. As a result, individuals who were planning to conceive or were already pregnant might have turned more heavily to online channels to seek information related to pregnancy and maternal health. This behavioral shift could, if anything, lead to an increase in fertility-related search activity during lockdowns. Second, our treatment measure is defined at the city level based on “high-risk” classifications, which may not fully capture variation in exposure within cities. Consequently, less-affected areas within treated cities may dilute the average treatment effect, leading to an underestimation of the true behavioral response. Together, these factors imply that our estimated effect likely represents a lower bound. Both potential upward bias in the search index and downward misclassification of treatment exposure work against detecting stronger effects. Nonetheless, the consistent alignment between our findings and observed national birth trends affirms the validity of our approach and underscores the value of behavioral data for early detection of demographic change.

### 4.2 Assessing estimator validity: goodman-bacon decomposition

While the results in Table 3 indicate a significant negative effect of COVID-19 shocks on fertility-related search behavior, a potential limitation of the TWFE model warrants attention. When treatment timing varies across units, the TWFE estimator may be biased due to inappropriate comparisons between already-treated and not-yet-treated groups. As emphasized by (19), such “forbidden comparisons” can generate negative weights in the underlying decomposition, potentially attenuating or even reversing the true average treatment effect. This issue is particularly salient in the presence of treatment effect heterogeneity across time or units (56).

To evaluate the extent of this potential bias, we perform a Goodman-Bacon decomposition following (19). This method decomposes the overall TWFE estimate into a weighted average of all possible two-group, two-period DID comparisons, where each comparison receives a weight proportional to its contribution to the estimator. The decomposition distinguishes three types of comparisons between Treatment (T) versus Control Groups (C): (1) early-treated units versus later-treated units, (2) later-treated units versus earlier-treated units–the so-called forbidden comparisons, and (3) treated units versus never-treated units.

**Figure 1:**
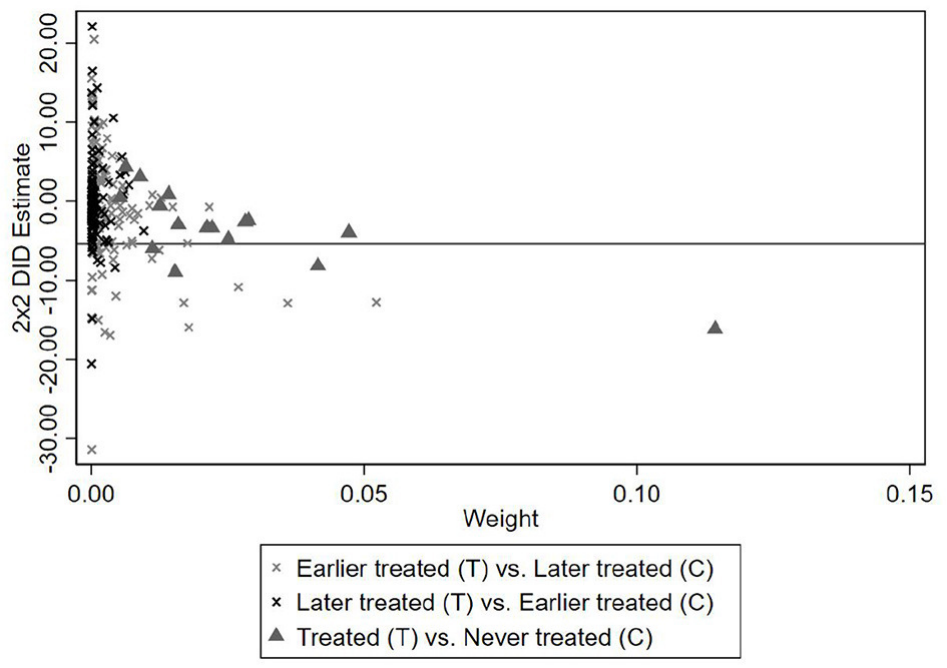
Goodman-bacon decomposition results.

Figure 1 visualizes the decomposition results, while Table 4 reports the weights and estimated effects associated with each comparison type. As shown in Table 4, the weight assigned to forbidden comparisons (“Later treated vs. Earlier treated”) is relatively small, accounting for only about 10% of the total. Moreover, the corresponding treatment effect estimates are closer to zero in magnitude, suggesting that any downward bias in the overall TWFE estimate is likely limited.

**Table 4:** Bacon decomposition: weight and average estimates.

**Comparison group: T vs. C**	**Weight**	**HCG**	**EDD**	**Progesterone**	**Pregnenolone**	**Index**
Earlier treated vs. later treated	0.479	-4.596	-8.290	-3.786	-2.885	-5.058
Later treated vs. earlier treated	0.103	-0.634	1.828	1.090	-2.517	-0.023
Treated vs. never treated	0.418	-6.208	-11.582	-5.598	-4.371	-7.098

This table reports the decomposition of the TWFE estimator following (19).

Taken together, the decomposition results indicate that bias due to staggered treatment timing is minimal in our context. As such, the baseline estimates in Table 3 can be interpreted as reliable approximations of the average treatment effect.

### 4.3 Imputation-based estimation

Although the Goodman-Bacon decomposition indicates that bias from forbidden comparisons is limited in our baseline TWFE model, we further implement an imputation-based estimator proposed by (20) to strengthen robustness. The core idea of this approach is to construct counterfactual outcomes for treated units based on observations from units that are not yet treated or are never treated. Specifically, for each treated city and time period, we impute its untreated potential outcome and compute the treatment effect as the difference between observed and imputed outcomes. By aggregating these unit-time level effects, we obtain an estimate of the average treatment effect on the treated (ATT). This method avoids problematic comparisons involving already-treated units and is therefore more reliable in settings with staggered treatment adoption and heterogeneous treatment effects across cohorts. Compared to the TWFE estimator, the imputation approach is less susceptible to bias from dynamic treatment timing and offers greater credibility in causal inference.

Estimation results using the imputation-based method are reported in Table 5. The findings are consistent with the TWFE estimates in both sign and magnitude. In Column (5), the coefficient on *Shock* is estimated at −5.489 and is statistically significant at the 1% level, indicating that exposure to a COVID-19 shock reduced the daily composite fertility search index by approximately 5.5 points. Relative to the pre-treatment average of 65.4 for treated cities (see Table 2), this corresponds to a decline of about 8.4%.

**Table 5:** The impact of COVID-19 shocks on fertility-related search index: imputation-based estimation.

**Dependent variable**	**HCG**	**EDD**	**Progesterone**	**Pregnenolone**	**Index**
	**(1)**	**(2)**	**(3)**	**(4)**	**(5)**
Shock	-4.944***	-9.379***	-3.779***	-3.143*	-5.489***
	(1.769)	(2.519)	(1.390)	(1.619)	(1.787)
2019 city controls × Month FE	Yes	Yes	Yes	Yes	Yes
City FE	Yes	Yes	Yes	Yes	Yes
Month FE	Yes	Yes	Yes	Yes	Yes
Observations	10,656	10,656	10,656	10,656	10,656

Standard errors clustered at the city level are reported in parentheses. *p < 0.1, **p < 0.05, ***p < 0.01.

The results indicate a robust and statistically significant decline in fertility-related online search activity following COVID-19 exposure, even after controlling for city-specific characteristics, temporal shocks, and baseline covariates. The observed reduction mirrors national-level declines in birth rates, lending further credibility to the behavioral proxy and underscoring its utility for capturing early signals of population change^8^. Importantly, these results are also consistent across individual keyword regressions, reinforcing that the composite index captures a stable signal rather than being unduly influenced by any particular term. This enhances our confidence that the observed behavioral response reflects a broad-based fertility adjustment rather than an artifact of keyword selection.

### 4.4 Event study

The identification strategy of the DID framework rests on the parallel trends assumption, which states that, in the absence of treatment, treated and control groups would have followed similar outcome trajectories over time. Validating this assumption is essential for attributing post-treatment divergence to the causal effect of COVID-19 shocks, rather than to pre-existing differences or differential trends.

To test the parallel trends assumption and to explore the dynamic evolution of treatment effects, we implement an event study specification. Building on the specification in Equation 1, we estimate the model specified in Equation 2:


(2) $$\begin{array}{c} Y_{ct}=\alpha +\underset{k\neq -1}{\sum }\beta_{k}{\text{Shock}}_{ct}^{k}+X_{c}\mu_{t}+\lambda_{c}+\mu_{t}+\epsilon_{ct} \end{array}$$

where *Y*_*ct*_ denotes the fertility-related outcome for city *c* at time *t*, as defined in Panel A of Table 1. The term $Shock_{ct}^{k}$ is a set of event-time indicators that equal 1 if city *c* is *k* months relative to its treatment onset, and 0 otherwise. The month immediately preceding treatment (*k* = −1) is omitted as the reference category. The coefficients β_*k*_ trace the dynamic treatment effects over time, relative to this baseline period. Consistent with the baseline model, we include interactions between 2019 city-level covariates *X*_*c*_ and time fixed effects μ_*t*_, allowing the influence of pre-pandemic characteristics to vary flexibly over time. City fixed effects (λ_*c*_) control for time-invariant unobserved heterogeneity, while time fixed effects (μ_*t*_) account for nationwide shocks common to all cities. The error term ϵ_*ct*_ captures idiosyncratic variation. Standard errors are clustered at the city level throughout to account for serial correlation within cities.

For empirical implementation, we define the event window to span from 47 months before to 35 months after the treatment. To enhance readability in the graphical display, periods earlier than *k* = −30 and later than *k* = 20 are grouped into two aggregated bins. We then plot the estimated β_*k*_ coefficients and their 95% confidence intervals to assess the validity of the parallel trends assumption and to examine the dynamic evolution of treatment effects.

**Figure 2:**
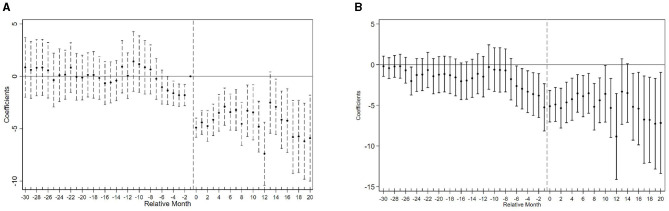
Event study estimates of COVID-19 shocks on fertility search index. **(A)** Two-way fixed effects estimation. **(B)** Imputation-based estimation.

Figure 2 displays the event study estimates of the COVID-19 shock's effect on the composite fertility search index. Panel (a) presents results based on the TWFE model, while Panel (b) shows estimates using the imputation-based estimator. The two specifications produce broadly similar patterns. Event study results for the four individual fertility indicators are presented in Section 2 of the Supplementary material and are consistent with the main findings for the composite index, and moreover demonstrate slightly more well-behaved pre-treatment trends.

Prior to the COVID-19 shocks, the trends in fertility-related search behavior between treated and control cities appear broadly parallel. Specifically, for months earlier than *k* = −4, the estimated β_*k*_ TWFE coefficients are close to zero and statistically insignificant. However, in the four months immediately preceding treatment, a downward trend begins to emerge, suggesting possible anticipatory effects or pre-trends.

This deviation may stem from the nature of high-risk classifications, which were rarely assigned abruptly. Instead, cities typically transitioned into high-risk status following a gradual escalation of local case numbers. Furthermore, geographic proximity to earlier high-risk areas may have triggered spillover effects via population flows, shared economic networks, or heightened public vigilance, all of which could induce behavioral changes prior to formal designation. These dynamics may explain the mild divergence observed immediately before treatment.

Following treatment onset, the fertility search index declines sharply and persistently (Figure 2A), reflecting a strong behavioral response to the pandemic. While both estimators yield similar dynamics, the sharp drop at *k* = 0 in Panel (a) reflects TWFE's compression of effects due to staggered treatment. The imputation-based estimator in Panel (b), by contrast, isolates cohort-specific counterfactuals and shows a smoother adjustment path.

### 4.5 Placebo test

To further assess the possibility that the observed effects are driven by unobserved confounders rather than true treatment, we implement a placebo test using the restricted mixed placebo procedure for staggered DID designs, as proposed by (57). Specifically, in each placebo iteration, we randomly assign cities into placebo-treated and placebo-control groups on a monthly basis, preserving the original group sizes from the actual sample. For placebo-treated cities, a hypothetical treatment date is also randomly assigned. Using this artificial treatment structure, we re-estimate the DID model and repeat the process 500 times to generate a distribution of placebo treatment effects.

**Figure 3:**
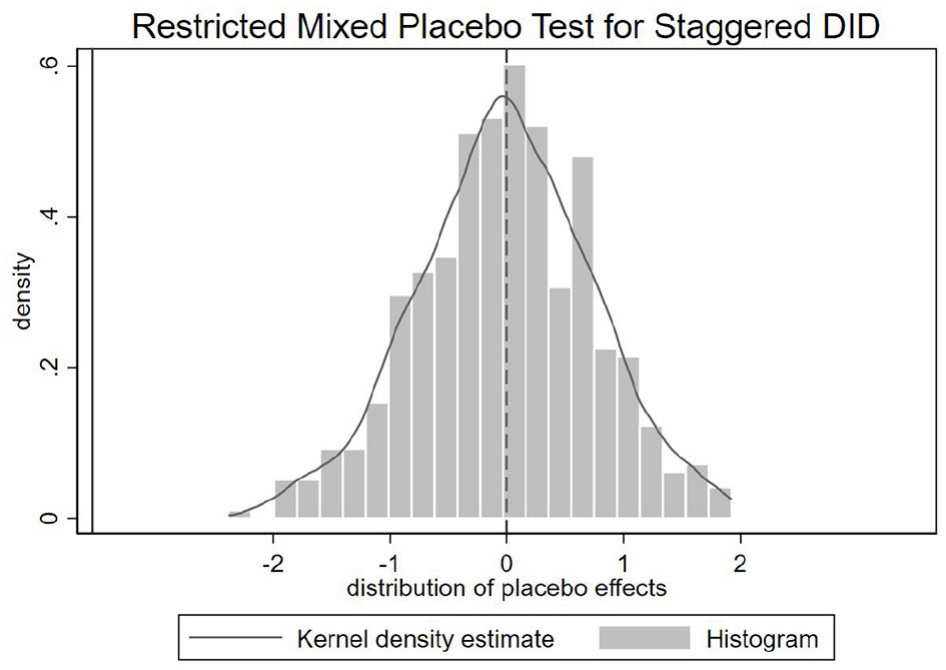
Placebo test results using the fertility search index.

Figure 3 presents the placebo test results using the composite fertility search index (*Index*) as the outcome variable. The solid vertical line marks the actual estimated treatment effect, which lies far outside the simulated distribution of placebo estimates. This indicates that the observed negative impact of COVID-19 shocks on fertility-related search behavior is unlikely to be driven by random variation or spurious correlations. These results further reinforce the credibility of our identification strategy. Placebo test results for individual fertility indicators are provided in Section 3 of the Supplementary material, showing broadly consistent patterns with the results based on the overall index.

### 4.6 Additional robustness checks

To further assess the robustness of our main findings, we conduct a series of supplementary analyses leveraging alternative specifications, variable constructions, and treatment definitions. These robustness checks fall into five main categories. First, we adopt a more exogenous treatment timing by defining the COVID-19 outbreak as a nationwide shock starting in January 2020, mitigating concerns over staggered or endogenous exposure. Second, we re-estimate our models using higher-frequency Baidu Index data–at weekly and daily resolutions–to ensure our results are not artifacts of temporal aggregation. Third, we test an alternative exposure definition that relaxes the 14-day high-risk requirement to capture earlier behavioral responses. Fourth, we construct multiple variants of the composite fertility index by sequentially excluding individual keywords to verify that our results are not driven by any single search term. Finally, we address potential confounding from differential local economic impacts by controlling for the monthly change ratio of the Gaode Mobility Index, a proxy for within-city changes in economic activity and policy restrictions.

Across all these exercises, the estimated effects of COVID-19 exposure on fertility-related search activity remain negative, statistically significant, and consistent in magnitude. This convergence across diverse empirical strategies provides strong evidence that our main results are not sensitive to modeling assumptions, data frequency, treatment timing, or variable definitions.

#### 4.6.1 Alternative treatment definition: COVID-19 outbreak as an exogenous shock

To mitigate identification concerns associated with staggered and endogenous treatment timing, we complement our main analysis with a standard DID approach using a more exogenous treatment definition: the onset of the COVID-19 pandemic in early 2020. Specifically, we treat January 2020 (2020M1) for the monthly specification and the first quarter of 2020 (2020Q1) for the quarterly specification as a uniform shock affecting all cities simultaneously, thereby assigning a common treatment onset across the sample. Unlike the high-risk designation, which typically followed gradual local case accumulation or spillovers from nearby regions, this approach applies a uniform treatment onset to all cities. Since no cities were affected before 2020Q1, the year 2019 serves as a consistent and credible pre-treatment baseline.

We implement this approach using a monthly panel covering 2019M1 to 2022M12 and estimate the following standard DID model:


(3) $$\begin{array}{c} Y_{ct}=\alpha +\beta \cdot Shock_{c}\times Post_{t}+X_{c}\mu_{t}+\lambda_{c}+\mu_{t}+\epsilon_{ct} \end{array}$$

where *Shock*_*c*_ is a binary indicator equal to 1 for cities ever exposed to COVID-19 during the sample period, and 0 otherwise. *Post*_*t*_ equals 1 for months from 2020M1 onward, and 0 for months in 2019. All other variables follow the baseline specification.

To verify the robustness of this monthly specification, we also replicate the analysis using a quarterly panel spanning 2019Q1 to 2022Q4. In this version, *Post*_*t*_ equals 1 for quarters from 2020Q1 onward, and 0 for quarters in 2019, following the same model structure.

Estimation results are reported in Table 6, with Panel A presenting the results based on monthly data and Panel B based on quarterly data. Consistent with our main findings (Table 3), we observe significant post-treatment declines in fertility-related search activity among treated cities. However, the effect sizes are somewhat smaller than those based on the imputation estimator (Table 5).

**Table 6:** COVID-19 outbreak as an exogenous shock: standard DID analysis.

**Dependent variable**	**HCG**	**EDD**	**Progesterone**	**Pregnenolone**	**Index**
	**(1)**	**(2)**	**(3)**	**(4)**	**(5)**
**Panel A: monthly Baidu Index data**					
Shock × Post	-2.913**	-6.297***	-2.419**	-2.177**	-3.518***
	(1.134)	(1.897)	(1.106)	(1.101)	(1.156)
Constant	60.121***	52.408***	66.920***	53.748***	58.366***
	(0.598)	(1.000)	(0.583)	(0.580)	(0.609)
2019 city controls × Month FE	Yes	Yes	Yes	Yes	Yes
City FE	Yes	Yes	Yes	Yes	Yes
Month FE	Yes	Yes	Yes	Yes	Yes
Observations	10,656	10,656	10,656	10,656	10,656
Adjusted *R*^2^	0.971	0.944	0.971	0.959	0.976
**Panel B: quarterly Baidu Index data**					
Shock × Post	-2.911*	-6.287***	-2.432*	-2.170*	-3.516**
	(1.135)	(1.899)	(1.109)	(1.103)	(1.158)
Constant	60.151***	52.401***	66.948***	53.760***	58.382***
	(0.598)	(1.001)	(0.584)	(0.581)	(0.610)
2019 city controls × Quarter FE	Yes	Yes	Yes	Yes	Yes
City FE	Yes	Yes	Yes	Yes	Yes
Quarter FE	Yes	Yes	Yes	Yes	Yes
Observations	3,552	3,552	3,552	3,552	3,552
Adjusted *R*^2^	0.980	0.955	0.980	0.968	0.979

Standard errors clustered at the city level are reported in parentheses. *p < 0.1, **p < 0.05, ***p < 0.01.

This attenuation is expected: by assigning the same treatment time to all exposed cities regardless of when they were actually affected, this specification averages over heterogeneous treatment timings. As a result, cities impacted later in the pandemic dilute the estimated average effect, biasing results toward zero relative to true causal impacts.

**Figure 4:**
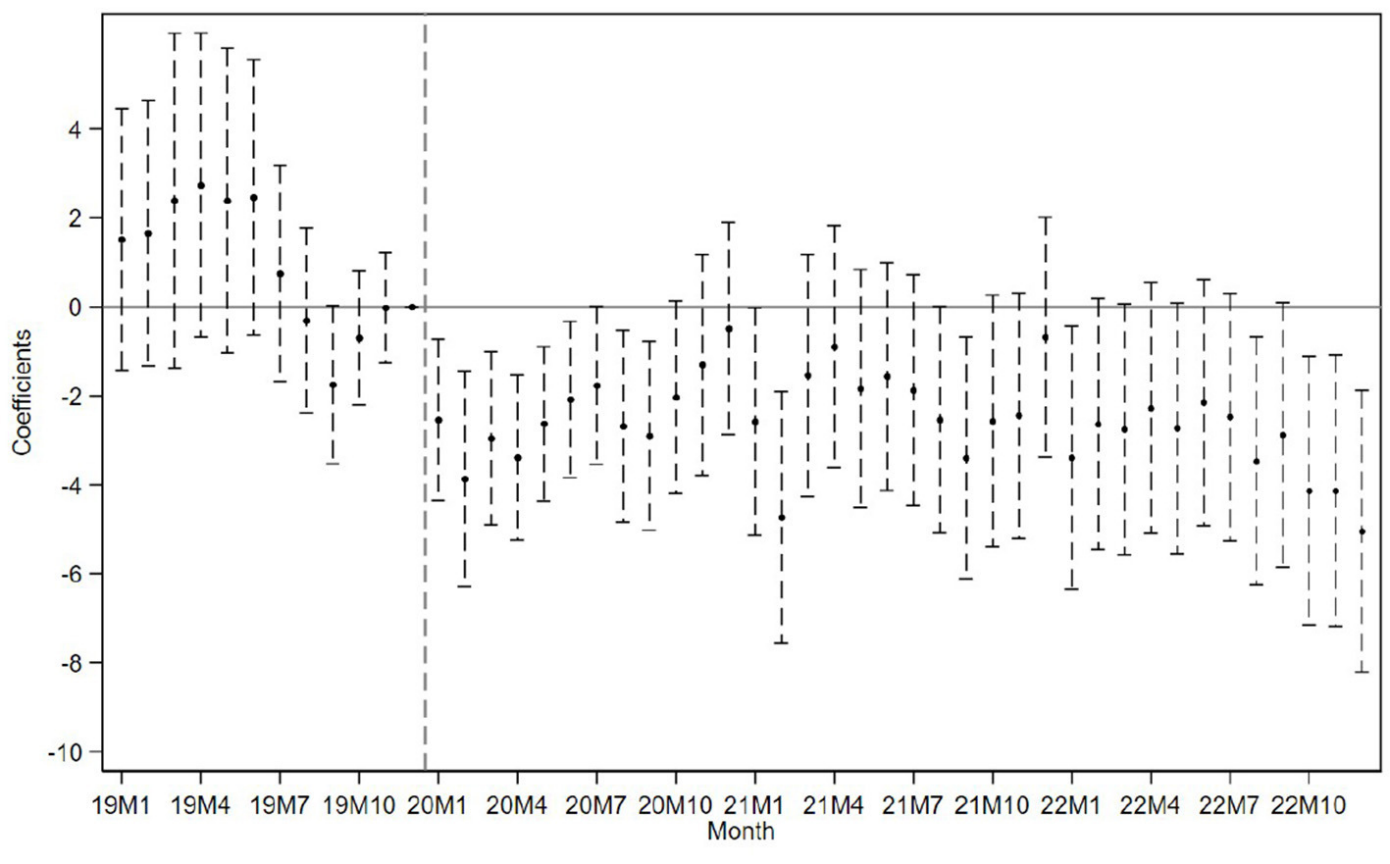
Event study using monthly data with exogenous shock defined by outbreak onset timing.

**Figure 5:**
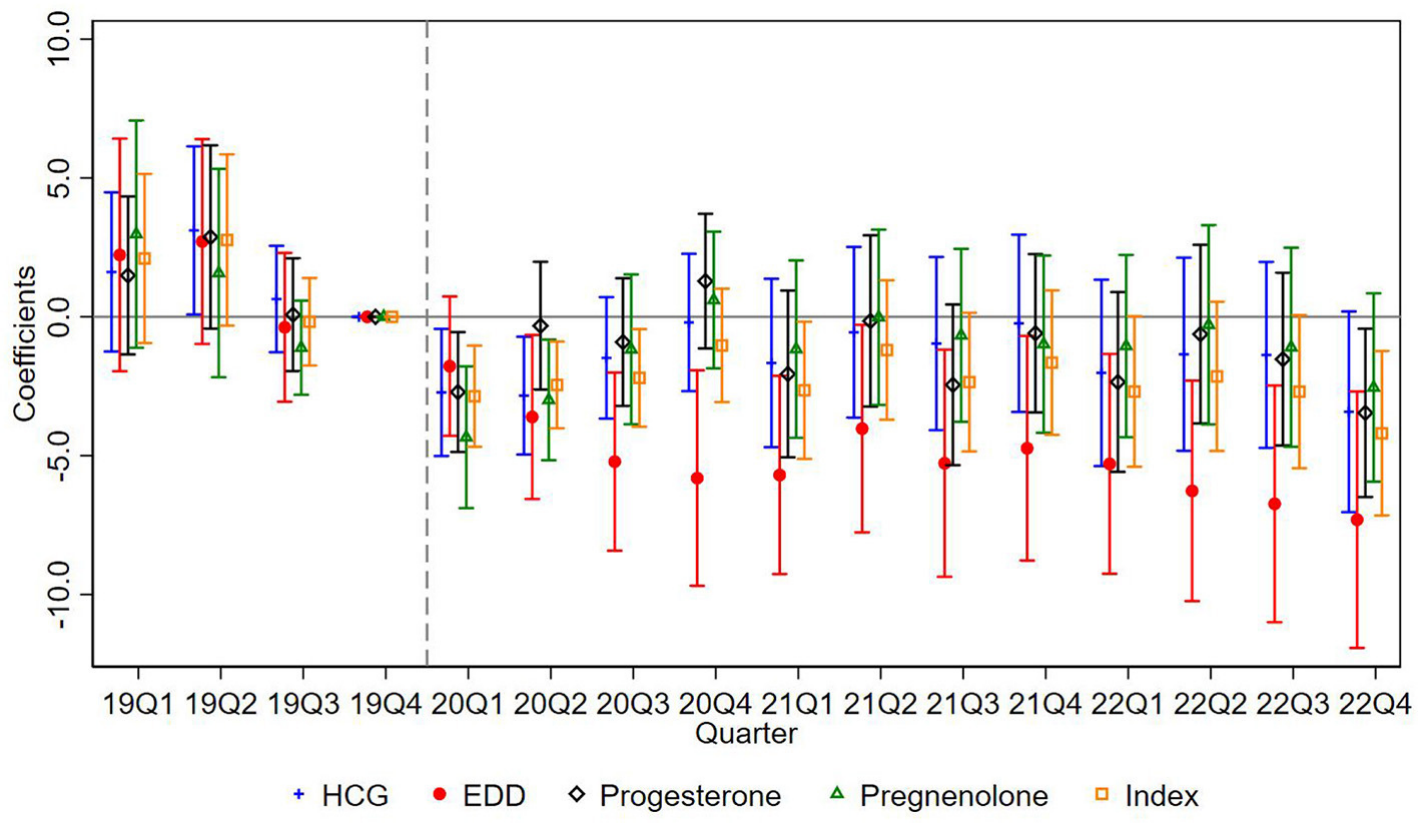
Event study using quarterly data with exogenous shock defined by outbreak onset timing.

In addition to regression estimates, we present visual evidence from corresponding event study analyses. Figure 4 plots the dynamic effects using monthly data, displaying the full window from 2019M1 to 2022M12 (i.e., January 2019 to December 2022). For clarity of presentation, we report only the composite index in the main figure, while the trajectories of the four individual keywords closely mirror that of the index and are available upon request. Figure 5 presents results based on quarterly data from 2019Q1 to 2022Q4 (i.e., the first quarter of 2019 to the fourth quarter of 2022), displaying dynamic treatment effects separately for all four individual keywords as well as the composite index.

Importantly, across both specifications–monthly and quarterly—we no longer observe the pre-treatment dip that appeared in our baseline event study using staggered high-risk exposure. This supports the view that the dip may have been driven by endogenous treatment timing or anticipatory behavior, both of which are mitigated under this exogenous shock specification.

#### 4.6.2 Alternative temporal resolution: weekly and daily Baidu Index

To further test the robustness of our findings, we complement the monthly and quarterly analysis with higher-frequency data. Specifically, we re-estimate the baseline model using weekly and daily Baidu Index data from January 1, 2019, to December 31, 2022. This exercise serves two purposes: first, to verify that the observed treatment effects are not artifacts of temporal aggregation; and second, to ensure that short-term fluctuations do not materially distort the results.

Estimation results are presented in Table 7. Panel A reports the estimates using weekly Baidu Index data, showing consistently negative and statistically significant effects across all fertility-related outcomes. The magnitudes are comparable to those from the baseline monthly specification, indicating that the estimated impacts are robust to moderate changes in temporal resolution. Panel B displays results based on daily data. With the exception of *Pregnenolone*, the coefficients remain negative and statistically significant, and their sizes are broadly similar to those from the weekly and monthly models. These findings suggest that the observed decline in fertility-related search behavior is not an artifact of time aggregation or short-term data volatility.

**Table 7:** Robustness checks using weekly and daily baidu index data.

**Dependent variable**	**HCG**	**EDD**	**Progesterone**	**Pregnenolone**	**Index**
	**(1)**	**(2)**	**(3)**	**(4)**	**(5)**
**Panel A: weekly Baidu Index data**					
Shock	-2.975***	-5.640***	-2.031**	-1.850*	-3.225***
	(1.131)	(1.582)	(0.926)	(1.058)	(1.080)
Constant	59.078***	49.991***	65.966***	52.896***	57.031***
	(0.186)	(0.260)	(0.152)	(0.174)	(0.178)
2019 city controls × Week FE	Yes	Yes	Yes	Yes	Yes
City FE	Yes	Yes	Yes	Yes	Yes
Week FE	Yes	Yes	Yes	Yes	Yes
Observations	46,398	46,398	46,398	46,398	46,398
Adjusted *R*^2^	0.940	0.909	0.940	0.926	0.965
**Panel B: daily Baidu Index data**					
Shock	-2.907**	-5.623***	-1.984**	-1.660	-3.144***
	(1.139)	(1.597)	(0.932)	(1.059)	(1.085)
Constant	59.069***	49.968***	65.966***	52.864***	57.015***
	(0.186)	(0.260)	(0.152)	(0.173)	(0.177)
2019 city controls × Day FE	Yes	Yes	Yes	Yes	Yes
City FE	Yes	Yes	Yes	Yes	Yes
Day FE	Yes	Yes	Yes	Yes	Yes
Observations	324,342	324,342	324,342	324,342	324,342
Adjusted *R*^2^	0.769	0.717	0.770	0.744	0.904

Standard errors clustered at the city level are reported in parentheses. *p < 0.1, **p < 0.05, ***p < 0.01.

#### 4.6.3 Alternative treatment timing: weekly data with revised exposure definition

To further address concerns about anticipatory behavior and endogenous treatment timing, we conduct an additional robustness check using an alternative definition of the treatment onset.

In our main analysis, a city is considered “treated” if it was either designated as high-risk for at least 14 consecutive days or subjected to a citywide lockdown. In this supplementary specification, we relax the 14-day threshold and define a city as treated if it was ever designated as high-risk or placed under lockdown, regardless of duration. This revision effectively moves the treatment timing forward by approximately two weeks, and thus allows us to capture behavioral responses that may have occurred at earlier signals of local outbreaks.

We apply this new treatment definition to both the monthly and weekly datasets. As expected, the monthly estimates remain nearly unchanged due to limited sensitivity to a two-week shift. Therefore, we focus on the weekly results, which provide greater temporal resolution to evaluate early behavioral reactions. The regression results (available upon request) remain robust and substantively similar: fertility-related search activity declines significantly following treatment, with no pronounced divergence prior to exposure.

**Figure 6:**
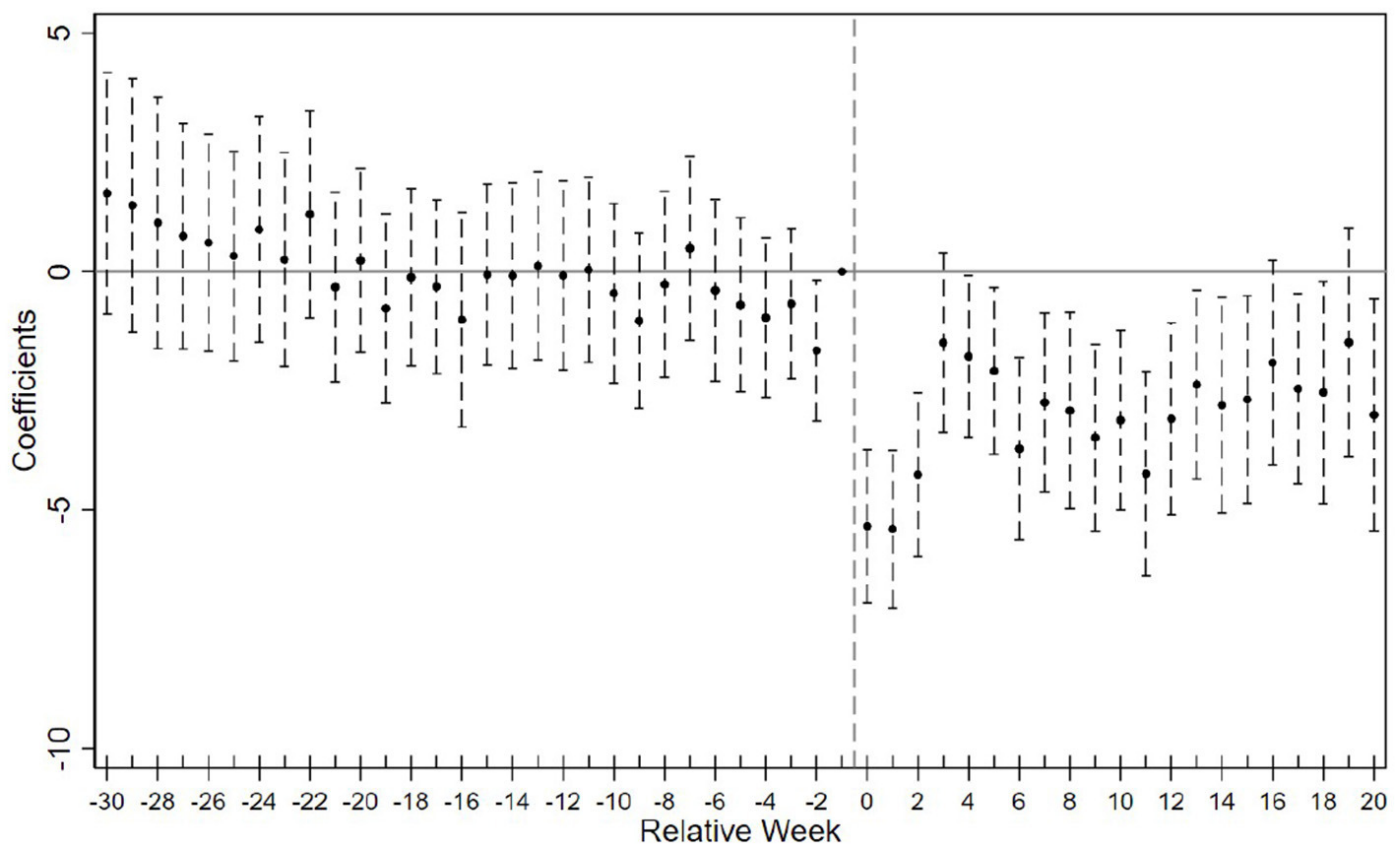
Event study estimates of COVID-19 shocks on fertility search index using weekly data and revised treatment definition.

The event study plot in Figure 6 shows a clear post-treatment drop in search behavior and no evidence of a systematic pre-treatment dip, except for a minor deviation observed in the second week before treatment, which likely reflects localized noise or early public concern.

These results reinforce our interpretation of the COVID-19 outbreak as a plausibly exogenous shock. The adjusted treatment timing alleviates concerns about anticipatory behavior driving pre-trend violations, and confirms that our main findings are not artifacts of treatment definition.

#### 4.6.4 Alternative dependent variable construction: leave-one-out index

To assess the robustness of our findings to keyword selection, we perform a sensitivity analysis using alternative index constructions. Specifically, we create four variations of the composite fertility search index by excluding one keyword at a time and re-estimate the main regressions using each variant. This leave-one-out strategy helps evaluate whether our main results are driven by any single keyword.

Estimation results are summarized in Table 8. Across all four alternative indices, the estimated treatment effects remain negative, statistically significant, and similar in magnitude to the original composite index. This consistency suggests that no single keyword disproportionately drives the main findings, and that our index captures a stable and broad-based signal of fertility-related behavior.

**Table 8:** Alternative dependent variable construction: leave-one-out index.

	**Index_wo_HCG**	**Index_wo_EDD**	**Index_wo_Prog**	**Index_wo_Preg**	**Index**
	**(1)**	**(2)**	**(3)**	**(4)**	**(5)**
**Panel A: two-way fixed effects**					
Shock	-3.610***	-2.787***	-3.854***	-3.849***	-3.546***
	(1.082)	(1.018)	(1.143)	(1.095)	(1.074)
Constant	56.430***	59.448***	54.088***	58.454***	57.114***
	(0.184)	(0.173)	(0.194)	(0.186)	(0.182)
2019 city controls × Month FE	Yes	Yes	Yes	Yes	Yes
City FE	Yes	Yes	Yes	Yes	Yes
Month FE	Yes	Yes	Yes	Yes	Yes
Observations	10,656	10,656	10,656	10,656	10,656
Adjusted *R*^2^	0.973	0.976	0.973	0.976	0.976
**Panel B: imputation-based estimation**					
Shock	-5.681***	-4.099**	-5.921***	-6.117***	-5.489***
	(1.819)	(1.604)	(1.914)	(1.831)	(1.787)
2019 city controls × Month FE	Yes	Yes	Yes	Yes	Yes
City FE	Yes	Yes	Yes	Yes	Yes
Month FE	Yes	Yes	Yes	Yes	Yes
Observations	10,656	10,656	10,656	10,656	10,656

Standard errors clustered at the city level are reported in parentheses. *p < 0.1, **p < 0.05, ***p < 0.01. The composite *Index* is constructed as the average of four fertility-related keywords: *HCG, EDD, Progesterone*, and *Pregnenolone*. Each column labeled *Index_wo_X* corresponds to an alternative version of the index excluding keyword *X* (e.g., *Index_wo_HCG* is the average of the remaining three terms). The notation “wo” stands for “without.” For brevity, *Progesterone* and *Pregnenolone* are abbreviated as *Prog* and *Preg*, respectively, in column labels.

#### 4.6.5 Controlling for local economic activity: Gaode Mobility change ratio

To account for differential local economic impact during the pandemic, we control for the monthly change ratio of the Gaode Mobility Index. This variable captures relative within-city changes in mobility intensity–serving as a proxy for evolving local economic activity and policy stringency.

Table 9 presents monthly regression results with and without controlling for the Gaode Mobility change ratio (Panels A and B, respectively). Across specifications, the treatment effect remains significantly negative and of similar magnitude. These results reinforce that our findings are not driven by local economic fluctuations or mobility trends, but instead reflect behavioral responses to COVID-19 risk exposure.

**Table 9:** Robustness checks controlling for the change ratio of gaode mobility index.

**Dependent variable**	**HCG**	**EDD**	**Progesterone**	**Pregnenolone**	**Index**
	**(1)**	**(2)**	**(3)**	**(4)**	**(5)**
**Panel A: controlling for mobility change ratio**					
Shock	-3.681***	-5.891***	-2.546***	-2.442**	-3.745***
	(1.114)	(1.482)	(0.923)	(1.036)	(1.049)
Mobility change ratio	-0.367	1.360***	-0.093	-0.419	0.126
	(0.289)	(0.277)	(0.248)	(0.281)	(0.206)
Constant	60.985***	50.794***	67.900***	54.301***	58.546***
	(0.197)	(0.261)	(0.166)	(0.186)	(0.186)
2019 city controls × Month FE	Yes	Yes	Yes	Yes	Yes
City FE	Yes	Yes	Yes	Yes	Yes
Month FE	Yes	Yes	Yes	Yes	Yes
Observations	9,870	9,870	9,870	9,870	9,870
Adjusted *R*^2^	0.972	0.946	0.972	0.960	0.977
**Panel B: without controlling for mobility change ratio (same sample)**					
Shock	-3.687***	-5.871***	-2.547***	-2.448**	-3.743***
	(1.114)	(1.485)	(0.924)	(1.037)	(1.050)
Constant	60.949***	50.924***	67.891***	54.261***	58.559***
	(0.195)	(0.259)	(0.161)	(0.183)	(0.183)
2019 city controls × Month FE	Yes	Yes	Yes	Yes	Yes
City FE	Yes	Yes	Yes	Yes	Yes
Month FE	Yes	Yes	Yes	Yes	Yes
Observations	9,870	9,870	9,870	9,870	9,870
Adjusted *R*^2^	0.972	0.946	0.972	0.960	0.977

Standard errors clustered at the city level are reported in parentheses. *p < 0.1, **p < 0.05, ***p < 0.01.

### 4.7 Heterogeneity analysis

Substantial variation in economic development, urbanization, and demographic composition across Chinese cities may have shaped local fertility responses to COVID-19. To investigate this, we conduct a series of heterogeneity analyses aligned with our theoretical shypotheses.

**Figure 7:**
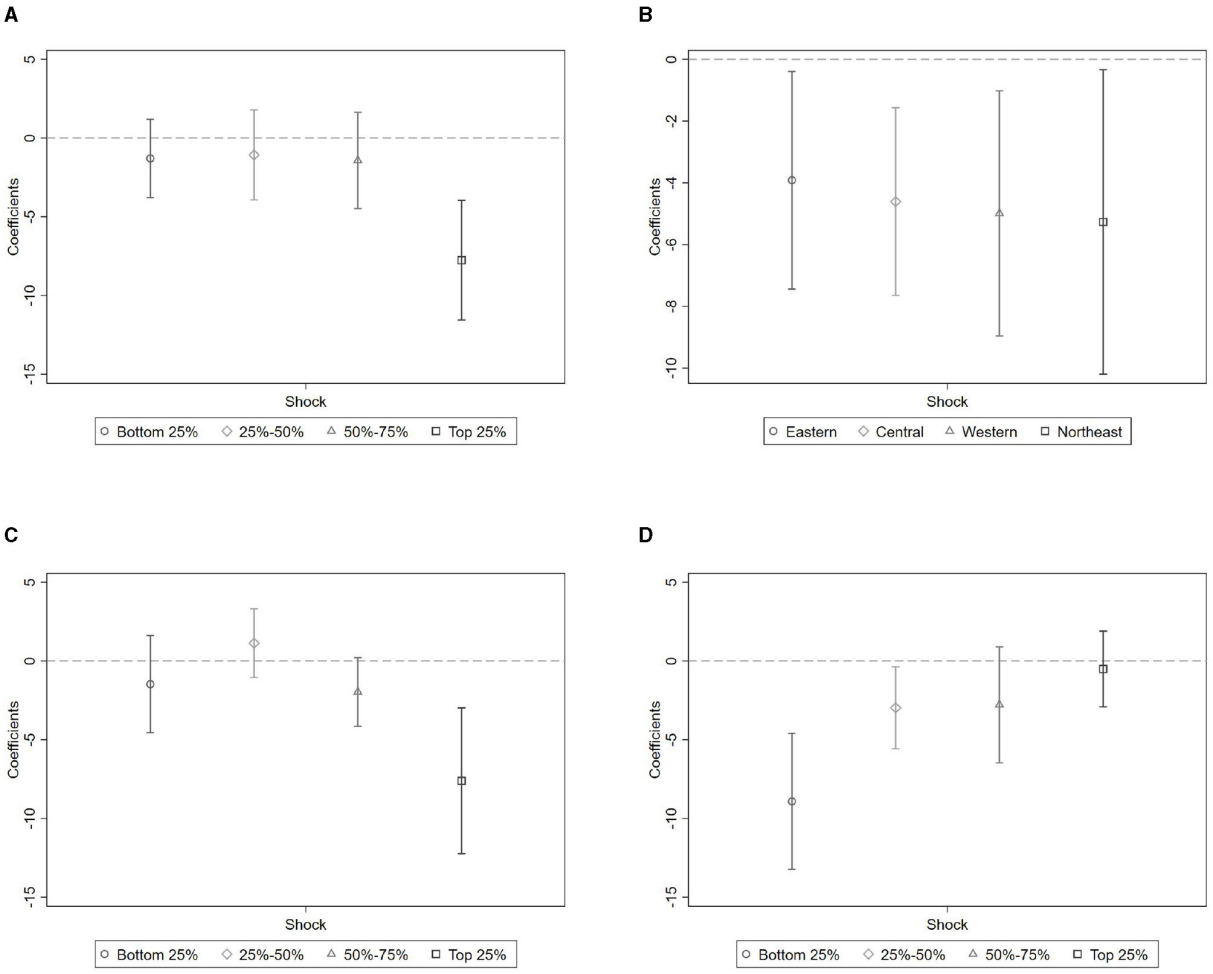
Heterogeneous effects by socioeconomic characteristics. **(A)** Economic development. **(B)** Region. **(C)** Urbanization level. **(D)** Demographic structure (male-to-female ratio).

We begin with economic development. Cities are divided into quartiles based on pre-pandemic GDP per capita. As shown in Figure 7A and Panel A of Table 10, significant negative effects are concentrated in the top quartile. This supports Hypothesis 2.1 and suggests that more developed cities—where fertility costs are higher and childcare systems more disrupted–experienced greater behavioral responses.

**Table 10:** Heterogeneity analysis.

	**(1)**	**(2)**	**(3)**	**(4)**
**Panel A: per capita GDP**				
	Bottom 25%	25–50%	50–75%	Top 25%
Shock	-1.297	-1.074	-1.426	-7.762***
	(1.488)	(1.709)	(1.827)	(2.270)
Constant	33.568***	43.521***	50.448***	101.495***
	(0.160)	(0.203)	(0.334)	(0.615)
2019 city controls × Month FE	Yes	Yes	Yes	Yes
City FE	Yes	Yes	Yes	Yes
Month FE	Yes	Yes	Yes	Yes
Observations	2,640	2,688	2,688	2,640
Adjusted *R*^2^	0.922	0.948	0.963	0.980
**Panel B: region**				
	Eastern	Central	Western	Northeast
Shock	-3.916*	-4.609**	-4.991**	-5.267*
	(2.115)	(1.823)	(2.372)	(2.864)
Constant	73.420***	55.763***	44.833***	40.246***
	(0.317)	(0.520)	(0.187)	(0.255)
2019 city controls × Month FE	Yes	Yes	Yes	Yes
City FE	Yes	Yes	Yes	Yes
Month FE	Yes	Yes	Yes	Yes
Observations	3,504	3,408	2,688	1,056
Adjusted *R*^2^	0.984	0.960	0.971	0.965
**Panel C: urbanization**				
	Bottom 25%	25–50%	50–75%	Top 25%
Shock	-1.472	1.131	-1.968	-7.616***
	(1.845)	(1.304)	(1.304)	(2.769)
Constant	34.229***	45.689***	50.854***	97.150***
	(0.244)	(0.194)	(0.204)	(0.668)
2019 city controls × Month FE	Yes	Yes	Yes	Yes
City FE	Yes	Yes	Yes	Yes
Month FE	Yes	Yes	Yes	Yes
Observations	2,688	2,640	2,640	2,688
Adjusted *R*^2^	0.947	0.945	0.937	0.982
**Panel D: male to female ratio**				
	Bottom 25%	25–50%	50–75%	Top 25%
Shock	-8.917***	-2.974*	-2.785	-0.507
	(2.582)	(1.558)	(2.201)	(1.439)
Constant	85.131***	58.077***	55.188***	30.397***
	(0.485)	(0.312)	(0.328)	(0.205)
2019 city controls × Month FE	Yes	Yes	Yes	Yes
City FE	Yes	Yes	Yes	Yes
Month FE	Yes	Yes	Yes	Yes
Observations	2,688	2,640	2,640	2,688
Adjusted *R*^2^	0.980	0.970	0.969	0.915

Standard errors clustered at the city level are reported in parentheses. *p < 0.1, **p < 0.05, ***p < 0.01.

To assess whether these patterns reflect economic structure or cultural factors, we further stratify cities by macroregion. Figure 7B and Panel B of Table 10 show that treatment effects are similar across Eastern, Central, Western, and Northeastern China, implying that economic conditions rather than regional culture or policy explain the observed heterogeneity.

Next, we test for heterogeneity by urbanization level. As shown in Figure 7C and Panel C of Table 10, cities in the top quartile of urbanization experienced significantly larger fertility declines. This finding supports Hypothesis 2.2 and highlights the vulnerability of highly urbanized environments, where family planning is more sensitive to economic and service disruptions.

Lastly, we explore whether local gender composition moderates the treatment effect. Cities are grouped by male-to-female ratio. As shown in Figure 7D and Panel D of Table 10, fertility-related search activity declined most sharply in cities with relatively more women. This pattern supports Hypothesis 2.3, and aligns with the view that women not only bore disproportionate caregiving burdens during the pandemic, but also exercised stronger decision-making power in reproductive choices (42).

Taken together, the heterogeneity analysis reveals that the fertility impact of COVID-19 was strongest in economically advanced, highly urbanized cities with larger female populations. These environments concentrate structural constraints—such as higher opportunity costs, disrupted childcare systems, and intensified caregiving expectations—that likely amplified pandemic-induced delays in childbearing.

These findings underscore the importance of locally targeted fertility support policies. In particular, interventions should focus on economically developed and urbanized areas with higher female population shares. Policy tools may include subsidized childcare, expanded parental leave, employment protections for women, and structural reforms to reduce gender inequality in caregiving and labor market participation.

Overall, our analysis shows that COVID-19 shocks significantly suppressed fertility-related behavior across Chinese cities. The results are robust to alternative specifications, placebo simulations, and varying temporal resolutions. By combining methodological advances–such as the imputation estimator and event-study validation–with a rich heterogeneity analysis, we provide credible evidence of both the average effect and its variation across structural contexts.

## 5 Discussion

This study provides new empirical evidence that COVID-19 shocks significantly suppressed fertility-related behavior across Chinese prefecture-level cities. Using high-frequency search data as a proxy for fertility intentions, we find that exposure to pandemic disruptions led to a clear decline in online searches related to pregnancy. These effects are particularly pronounced in the immediate months following a city's designation as a high-risk area, suggesting a sharp behavioral response to acute uncertainty. The magnitude of the effect varies substantially across cities, with stronger declines observed in areas characterized by higher income, greater urbanization, and larger female population shares. These patterns underscore the role of structural and demographic context in mediating behavioral responses to large-scale public health shocks.

Our findings contribute to the growing literature on the demographic effects of public health crises. Whereas prior research has primarily relied on official birth statistics to document delayed fertility responses after events such as wars, recessions, or pandemics (10, 17, 26), our study shifts attention to behavioral intentions observed in near real time. This conceptual and methodological distinction is especially relevant in the context of fast-evolving crises like COVID-19, where early indicators are crucial for timely policy responses and institutional adaptation (16, 35). Moreover, in line with recent work linking fertility planning to macroeconomic expectations and individual risk perception (27), we show that online search behavior can serve as a sensitive, forward-looking proxy for reproductive decision-making–capturing anticipatory shifts well before they materialize in administrative records. Finally, our approach complements emerging micro-level findings on the role of gendered agency in fertility planning under uncertainty (42), underscoring that observed behavioral suppression may reflect not only institutional disruptions but also intra-household negotiation dynamics.

The observed heterogeneity in treatment effects highlights how city-level characteristics shape vulnerability to demographic disruption. In wealthier and more urbanized areas, where opportunity costs of childbearing are higher and family support networks weaker, fertility intentions are more susceptible to external shocks. Cities with larger female populations experienced sharper declines, reflecting not only disproportionate caregiving burdens during the pandemic but also greater female agency in reproductive decision-making (42). These findings suggest that behavioral fertility suppression—driven by heightened risk aversion, economic insecurity, and constrained caregiving capacity–may be particularly persistent in structurally strained contexts, dampening prospects for post-crisis recovery.

A plausible mechanism linking pandemic shocks to suppressed fertility intentions is increased economic pressure. A growing body of research shows that COVID-19 caused substantial economic disruptions, including income loss, unemployment, and heightened financial uncertainty, particularly among low-income and service-sector workers (58–60). (61) provide a comprehensive review of this literature, concluding that the pandemic led to widespread job losses and reduced consumer spending. These findings, together with broader evidence on fertility decline during past recessions (26, 62, 63), suggest that macroeconomic downturns and financial insecurity contribute to delayed or foregone childbearing. Recent COVID-specific studies further support this pattern, showing that economic strain and uncertainty were key drivers of short-term fertility declines across both high- and low-income settings (34, 64, 65).

These insights have important policy implications. First, short-term financial incentives alone are unlikely to offset pandemic-related fertility suppression. Policy reforms—especially in childcare, education affordability, and workplace protections for women–are essential. Second, behavioral data offer a valuable early warning tool. Real-time monitoring of fertility intentions can inform targeted, city-specific interventions in periods of crisis. Cities showing steep behavioral declines may benefit from stabilizing employment conditions, reducing uncertainty, and expanding mental health and caregiving support. Third, a long-term fertility strategy must address deeper institutional constraints: work-family conflict, gender inequality, and the precarity of youth labor markets.

While our empirical setting is grounded in the China context, the underlying question we address—how public health shocks alter fertility-related behavior—has broader global relevance. Across diverse institutional contexts, the pandemic disrupted reproductive decision-making by introducing heightened uncertainty, economic insecurity, and barriers to healthcare access. Our methodological approach—combining web search data with region-specific shock exposure—can be extended to other countries with similar digital and epidemiological infrastructure. For example, researchers in the U.S. or Europe could use Google Trends data on pregnancy-related keywords alongside county-level COVID-19 case counts or lockdown policies to examine parallel behavioral responses (49–51). These adaptations would allow for real-time tracking of fertility intentions in settings where official birth data are lagged or incomplete. However, caution is warranted when extrapolating our empirical conclusions to other countries. Differences in policy stringency, social norms, healthcare systems, and demographic structures may shape both exposure to public health shocks and the behavioral responses they elicit.

Several limitations should be noted. First, while we validate our fertility search index against official data, it remains an aggregate proxy and does not capture subgroup variation by age, marital status, or parity. Future research could link behavioral data with micro-level survey or administrative records to uncover more nuanced patterns. Second, although our empirical strategy exploits variation in COVID-19 shock exposure, we do not disentangle overlapping channels such as infection risk, lockdown policies, and economic disruptions. Identifying these mechanisms—perhaps through policy discontinuities or instrumental designs–remains an important direction. Third, our study focuses exclusively on China. Given cross-national differences in socioeconomic conditions and institutional contexts, the generalizability of our findings should be interpreted with caution. Nonetheless, the methodology we propose—combining digital behavioral data with staggered causal designs–offers a flexible framework that can be applied in other low- and middle-income countries facing similar data limitations, potentially enabling timely monitoring of fertility-related behaviors in data-scarce settings. Lastly, our keyword selection is based on a context-specific rather than a corpus-driven approach, which may omit other relevant pregnancy-related terms. Future research could employ corpus-based methods to systematically broaden the keyword set.

Taken together, our findings suggest that public health shocks can trigger immediate and uneven shifts in reproductive behavior. Real-time behavioral data can serve as a valuable complement to traditional demographic statistics, helping researchers and policymakers anticipate and respond to demographic risks in rapidly changing environments.

## 6 Conclusion

This study examines how large-scale public health disruptions influence fertility-related behavior, using the COVID-19 pandemic as a quasi-natural experiment across Chinese prefecture-level cities. Leveraging Baidu search data as a high-frequency proxy for reproductive intentions, we find that city-level pandemic shocks led to significant and persistent declines in fertility-related search activity. These effects were especially pronounced in more urbanized and economically developed areas, and in cities with larger female population shares–highlighting the role of structural and gendered constraints in shaping behavioral responses to crisis.

Our findings contribute to a growing body of health economics research that examines how uncertainty and institutional shocks affect individual decision-making. While previous studies have focused primarily on realized fertility outcomes, we demonstrate the value of digital behavioral signals as early indicators of demographic stress. In the context of fast-moving public health crises, such behavioral proxies can inform real-time policy responses–particularly in settings where official demographic data are delayed or incomplete.

These results have direct policy relevance. Fertility suppression during the pandemic was not uniform, but shaped by pre-existing inequalities in care burdens, employment conditions, and access to supportive infrastructure. Addressing these structural barriers is essential for designing effective, equity-focused fertility policies. Interventions should go beyond financial incentives to include expanded childcare services, gender-equal labor protections, and reductions in parenting-related costs–especially in cities where constraints are most binding.

Finally, our approach illustrates how digital trace data can support scalable population monitoring in low- and middle-income countries. As demographic challenges intensify, integrating high-frequency behavioral indicators into public health surveillance systems may help governments detect early warning signals, tailor policy interventions, and advance global goals related to health equity and sustainable population development.

## Data Availability

The original contributions presented in the study are included in the article/Supplementary material, further inquiries can be directed to the corresponding author.

## Footnotes

^1^See the Special report on China's population research from the Laboratory of Data Science and Business Intelligence website. Link: https://dsbi.swufe.edu.cn/info/1131/3821.htm, accessed May 8, 2025.

^2^See (8) and (9) for a broader overview of the pandemic's economic and behavioral impacts.

^3^All Baidu Index data were collected using Chinese-language keywords. Since exact one-to-one translations between Chinese and English medical terms may not always hold, we provide the original pinyin used for each term as follows: “HCG” (original English acronym), “Pregnenolone” (pinyin: *yuntong*), “Progesterone” (pinyin: *huangtitong*), and “EDD” (pinyin: *yuchanqi*).

^4^This distinguishes them from more emotionally or socially charged terms (e.g., “early pregnancy” or “miscarriage”), which may reflect media narratives or public debates unrelated to personal fertility behavior. Other biological markers, such as Estradiol, may also be relevant to early pregnancy, but they are not routinely tested or reported nationwide, limiting their consistency and comparability across settings.

^5^Source: https://index.baidu.com/v2/index.html#/.

^6^See the official guideline issued by the Joint Prevention and Control Mechanism of the State Council. Link: https://www.gov.cn/zhengce/2020-02/18/content_5480514.htm, accessed May 11, 2025.

^7^For background on the definition of “high-risk areas,” see the Baidu Encyclopedia entry: high-risk areas. The February 2020 risk classification guideline–defining high-risk areas as those with over 50 cumulative cases within 14 days–continued to serve as the policy basis for high-risk designations into 2021. See Sina Finance, Jan 2021 and Hubei Health Commission, Mar 2020, all accessed July 9, 2025.

^8^See the report by Yicai, titled “The Number of Births in China May Have Fallen by Over 10% in 2020, with Further Declines Expected Below 10 Million in Coming Years.” Link: https://www.yicai.com/news/100949424.html, accessed May 12, 2025.
