# Supplementary material for: The impact of public health shocks on fertility intentions: evidence from the COVID-19 pandemic in China
Source: Front Public Health. 2025 Aug 18;13:1631821. doi: 10.3389/fpubh.2025.1631821 (PMC12399519; doi:10.3389/fpubh.2025.1631821)
Supplement: Supplementary file 1 [file Data_Sheet_1.pdf]

Supplementary Material

1 VALIDATION OF THE BIRTH PROXY VARIABLE

Official birth statistics in China are typically published on an annual basis and often lack comprehensive coverage at the city level. During the COVID-19 pandemic, local statistical offices faced additional disruptions, resulting in further delays and data omissions. Consequently, relying solely on annual official birth rates would obscure short-term fertility dynamics and regional heterogeneity during public health emergencies. These limitations motivate the use of alternative high-frequency data sources.

To provide preliminary validation, we calculate the Pearson pairwise correlation coefficient between cities' average fertility search index and their officially reported resident birth rates in 2019, using data from the *China City Statistical Yearbook*. Due to data availability issues, birth rate information is missing for some cities. As a result, our final sample includes 192 out of the 222 cities used in the main analysis. The correlation coefficient is 0.194 and is statistically significant at the 1% level.

We further conduct Ordinary Least Squares (OLS) regressions to formally test the relationship between the fertility search index and official birth rates. The model specification is as follows:

$$BirthRate_i = \alpha + \beta Index_i + \gamma Controls_i + \epsilon_i \tag{S1}$$

where $BirthRate_i$ denotes the official resident birth rate for city i, $Index_i$ is the fertility search index, and $Controls_i$ include GDP per capita ($perGDP$), urbanization rate ($UrbanRate$), unemployment rate ($UnemploymentRate$), male-to-female ratio ($MFgender\_ratio$), and education level (Edu), consistent with the main analysis.

To enhance interpretability and facilitate coefficient comparison, both the birth rate and the fertility search index are rescaled using min-max normalization prior to regression. This transformation places the two variables on a comparable scale, allowing the magnitude of the estimated coefficient on the search index to more directly reflect the strength of their association.

Table S1 presents the regression results. The coefficient on the fertility search index is positive and statistically significant at the 1% level, even after controlling for key city-level covariates. This finding suggests that cities with higher levels of fertility-related search activity also tend to have higher actual birth rates, supporting the validity of the search index as a proxy for fertility behavior.

Table S1. Correlation between Birth Rate and Fertility Search Index

**Dependent Variable:** BIR	(1)	(2)
Index	0.151***	0.182***
	(0.056)	(0.058)
Constant	0.294***	-0.995***
	(0.014)	(0.338)
City Controls	No	Yes
Observations	192	192
Adjusted R^2	0.032	0.349

Note: Standard errors clustered at the city level are reported in parentheses. * $p < 0.1$, ** $p < 0.05$, *** $p < 0.01$.

To further enhance transparency, Table S2 reports the proportion of zero values for each search index across different temporal frequencies. While individual keywords (e.g., HCG, EDD) show a sizable share of zeros at the daily level, these proportions decrease substantially with aggregation. Notably, the share of

zero values for the composite $Index$ is only 1.2% at the monthly level—consistent with the data used in our main analysis. This result supports the reliability of the search index and alleviates concerns about data sparsity.

Importantly, we emphasize that no imputation, interpolation, or exclusion was performed on search index observations with zero values. All values, including zeros, were retained in the dataset to preserve the authenticity of the raw Baidu data.

While Baidu does not disclose the full details of its smoothing or filtering algorithm, our approach minimizes potential distortion by aggregating daily observations to higher temporal resolutions—specifically, monthly averages. As shown in Table S2, this aggregation dramatically reduces the proportion of zero values across all variables. For instance, although daily zeros are common (over 35% for some keywords), monthly zeros fall below 5% for all individual indices and to just 1.2% for the composite $Index$.

This aggregation strategy not only improves signal stability but also helps reduce the influence of any undocumented algorithmic smoothing by Baidu. Taken together, the low proportion of zeros in the final dataset—along with our transparent and conservative treatment of potential platform-induced artifacts—reinforces the credibility and reliability of our empirical analysis.

Table S2. Proportion of Zero Values in Search Indices by Frequency

Frequency	Variable	Zero Count	Total Observations	Proportion (%)
	HCG	116,787	324,342	36.01
	EDD	135,065	324,342	41.64
Daily	Progesterone	81,021	324,342	24.98
	Pregnenolone	117,080	324,342	36.10
	Index	40,435	324,342	12.47
	HCG	4,525	46,398	9.75
	EDD	6,532	46,398	14.08
Weekly	Progesterone	2,929	46,398	6.31
	Pregnenolone	4,672	46,398	10.07
	Index	1,595	46,398	3.44
	HCG	388	10,656	3.64
	EDD	515	10,656	4.83
Monthly	Progesterone	304	10,656	2.85
	Pregnenolone	377	10,656	3.54
	Index	128	10,656	1.20
	HCG	68	3,552	1.91
	EDD	74	3,552	2.08
Quarterly	Progesterone	46	3,552	1.30
	Pregnenolone	63	3,552	1.77
	Index	11	3,552	0.31

2 EVENT STUDY RESULTS FOR INDIVIDUAL KEYWORDS

Figure S1 presents the dynamic treatment effects for each of the four individual fertility-related search indices, estimated separately using the two-way fixed effects model and the imputation-based estimator.

The horizontal axis indicates event time k, defined as the number of months relative to the treatment onset, and the vertical dashed line marks the beginning of the COVID-19 shock.

Overall, the event study results closely mirror those reported in the main text for the composite index. Prior to the treatment, treated and control cities display broadly parallel trends across all four fertility-related outcomes. Following the COVID-19 shock, significant and persistent declines in search intensity are observed, confirming the negative impact of the pandemic on fertility-related online behavior.

However, there are some differences across different fertility proxies. In particular, the estimated effects for *EDD* remain significantly negative over an extended post-treatment period, showing little evidence of recovery. In contrast, the effects for *HCG*, *Progesterone*, and *Pregnenolone* gradually attenuate over time, with coefficients trending back toward zero.

This divergence likely reflects the timing differences inherent in fertility-related search behavior. Searches for *HCG*, *Progesterone*, and *Pregnenolone* are typically associated with early stages of pregnancy, whereas *EDD* queries occur later in the gestation process. The partial rebound in early-stage indicators suggests that some individuals resumed previously postponed fertility plans as public health measures took effect and uncertainty began to recede.

3 PLACEBO TESTS FOR INDIVIDUAL KEYWORDS

This appendix presents the placebo test results for each of the four individual fertility-related search indices: *HCG*, *EDD*, *Progesterone*, and *Pregnenolone*. Following the same procedure as in the main analysis, we perform 500 placebo replications for each outcome by randomly reassigning both treatment groups and treatment timing, using the restricted mixed placebo test framework.

Figure S2 displays the distributions of placebo treatment effects for each individual fertility indicator. In all cases, the actual estimated effect (shown as a solid vertical line) falls far outside the simulated placebo distributions. These results provide further evidence that the observed effects are not driven by random chance and reinforce the robustness of the baseline findings reported in the main text.

4 FIGURES

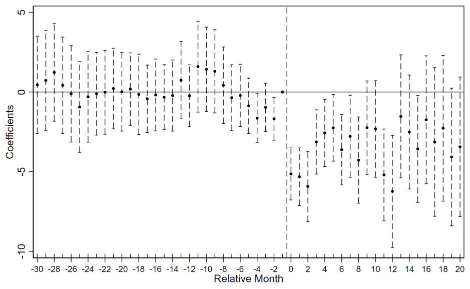

(a) TWFE - HCG

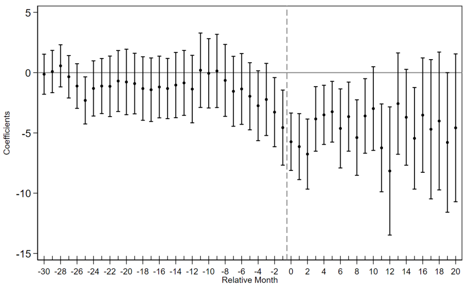

(b) Imputation - HCG

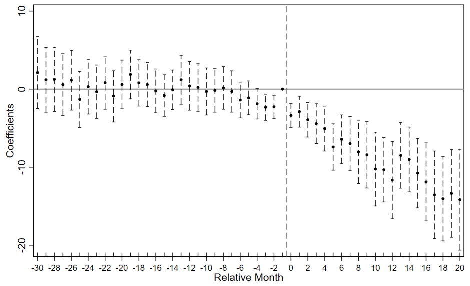

(c) TWFE - EDD

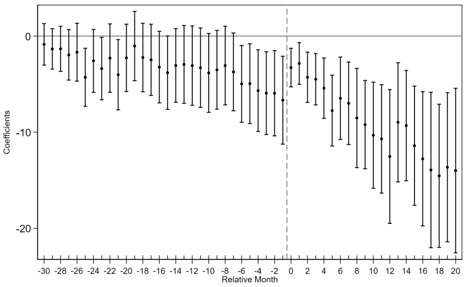

(d) Imputation - EDD

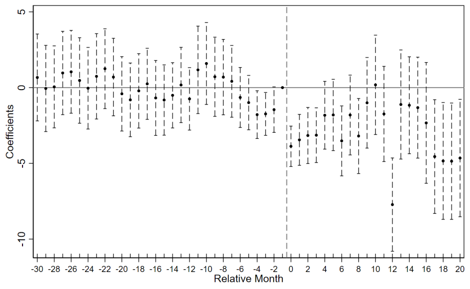

(e) TWFE - Progesterone

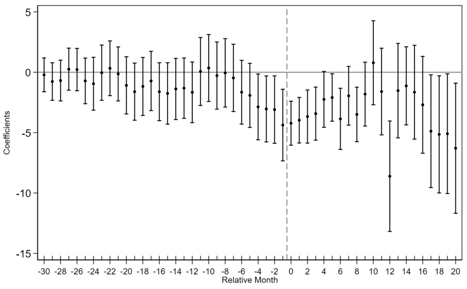

(f) Imputation - Progesterone

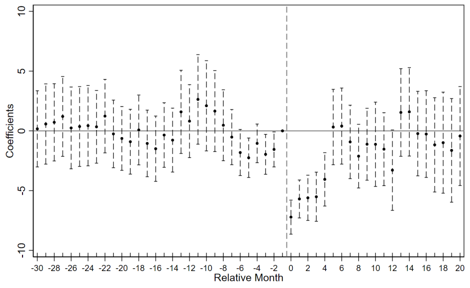

(g) TWFE - Pregnenolone

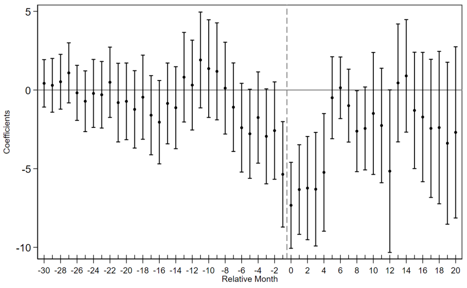

(h) Imputation - Pregnenolone

Figure S1: *Event study estimates.*

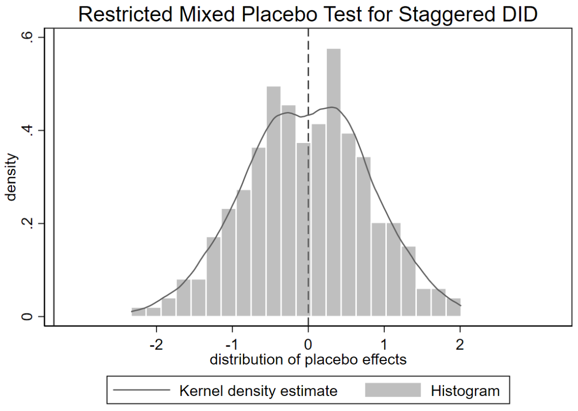

(a) HCG

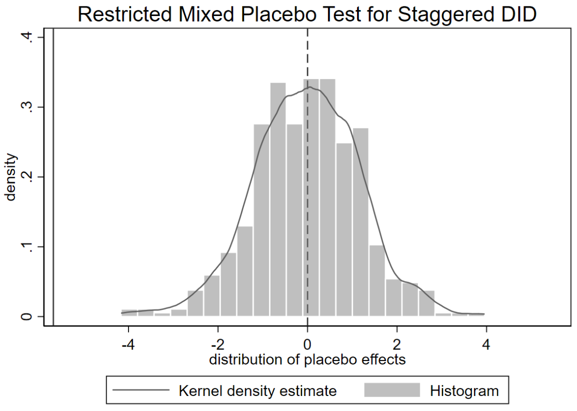

(b) EDD

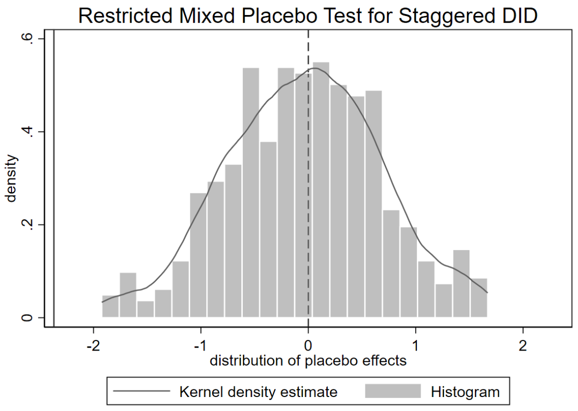

(c) Progesterone

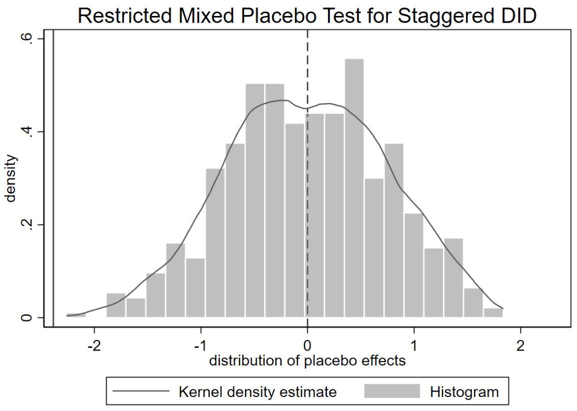

(d) Pregnenolone

Figure S2: *Placebo test results*
